# Effects of Cadmium on Lipid Storage and Metabolism in the Freshwater Crab *Sinopotamon henanense*

**DOI:** 10.1371/journal.pone.0077569

**Published:** 2013-10-10

**Authors:** Jian Yang, Dongmei Liu, Weixin Jing, Hans-Uwe Dahms, Lan Wang

**Affiliations:** 1 Laboratory of the Bio-effect and Molecular Mechanism of Classical Environmental Pollutants, School of Life Science, Shanxi University, Taiyuan, Shanxi Province, People’s Republic of China; 2 Department of Life Science, College of Natural Sciences, Sangmyung University, Seoul, South Korea; Texas Tech University, United States of America

## Abstract

Since environmental effects of molecular traits are often questioned we analyze here the molecular effects of cadmium (Cd) on lipid pathways and their effects on tissues development. Lipids are an important energy source for the developing embryo, and accumulate in the ovary and hepatopancreas of decapod crustaceans. The extend of Cd affecting lipid storage and metabolism, is studied here with the freshwater crabs *Sinopotamon henanense*. Crabs were exposed to water-born Cd at 1.45, 2.9, 5.8 mg/l for 10, 15, and 20 days. With significantly increased Cd accumulation in exposed crabs, lipid content in hepatopancreas and ovary showed a time-dependent and concentration-dependent reduction, being at least one of the reasons for a lower ovarian index (OI) and hepatopancreatic index (HI). After 10-day exposure increased triglyceride (TG) level in hemolymph and up-regulation of pancreatic lipase (PL) activity in the hepatopancreas suggested an increased nutritional lipid uptake. However, two processes led to lower lipid levels upon Cd exposure: an increased utilization of lipids and a down-regulated lipoprotein lipase (LPL) led to insufficient lipid transport. 10-day Cd exposure also triggered the production of β-nicotinamide adenine dinucleotide 2'-phosphate reduced tetrasodium salt hydrate (NADPH), as well as to the synthesis of adenosine triphosphate (ATP) and fatty acids. With increasing exposure time, the crabs at 15 and 20-day exposure contained less lipid and TG, suggesting that more energy was consumed during the exposure time. Meanwhile, the level of NADPH, ATP and the activity of PL, LPL, fatty acid synthase (FAS), acetyl-CoA carboxylase (ACC) activity was down-regulated suggesting an impairment of the crab metabolism by Cd in addition to causing a lower lipid level.

## Introduction

Due to anthropogenic sources, such as burning of coal in power stations and metal smelting, the trace heavy metal cadmium (Cd) is increasingly released to the environment [1,2]. Cd pollution is also frequently found in aquatic systems, such as rivers in China like the Yellow River and Yangtze River [3,4,5]. Cd could lead to acute or chronic intoxication of organisms and cause a variety of adverse effects, such as functional changes in the porcine renal proximal tubular epithelial cell line [6] and Cd-induced hepatopancreatic cell necrosis and apoptosis in crab [7]. In addition, Cd could cause an increase of reactive oxygen species that challenge the cellular antioxidant system, and lipid peroxidation in crustaceans [8,9,10]. The impairment of lipid induced by Cd has also been detected in the duck *Cairina moschata* and the European eel *Anguilla anguilla*, and might directly affect their migration [11,12]. Consequently，toxicity from Cd on aquatic organisms is becoming a global environmental problem that results in the disturbance of entire aquatic ecosystems [13,14]. The decline and extinction of species also occurred in some populations [15,16].

During crustacean ovarian development, especially in the phase of yolk accumulation, a large amount of lipid is stored in the ovary and hepatopancreas [17,18]. The lipid stored in eggs will be mobilized for energy production in the course of embryogenesis. Therefore, crustacean fecundity, survival, and egg growth are associated with lipid content and composition in the ovary [19,20]. The hepatopancreas, as a vital organ for crustaceans is used for lipid storage and synthesis and plays a major role in crustacean growth and reproduction. Lipids stored in the hepatopancreas are used for energy generation. They are also directly transferred to the ovary during ovarian development [21,22,23]. So did Chen et al. [24] observe a significant reduction of lipid content in the hepatopancreas of Chinese mitten crab in the stable phase of ovarian maturation. When environmental conditions deteriorate, such as during scarcity of food, energy is mainly derived from the consumption of lipids stored in crustacean tissues [25,26]. Undoubtedly, lipids are important for aquatic benthic animals, such as the freshwater crab, *Sinopotamon henanense*, which is widely distributed in the freshwater environments in the Yangtze River drainage, Huaihe River drainage and Yellow River Valley of China. This crab species lives at the interface of sediment and water column where Cd is deposited [27,28]. Despite the fact that crabs in their aquatic habitat are commonly subjected to multiple contamination sources, very few studies were focusing as yet on the effect of Cd on lipid storage and metabolism in brachyurans.

In the present study, we evaluate the possible impact of Cd on hepatopancreas and ovary, the two major tissues of Cd accumulation and lipid metabolism in the freshwater crab, *S. henanense* [24,29]. This also allowed insights to he mechanisms of decreased reproduction fitness and reduction of population size. The experimental Cd concentrations chosen were close to that of environmental Cd contaminations, such as Cd pollution incidents in Shaoguan city of Guangdong North River, 2005; and the Longjiang Cd pollution event in Hechi, Guangxi, 2011 [30]. 

In order to test the effects of Cd, we measured lipid content, triglyceride (TG) levels in hemolymph, as well as enzyme activity involved in lipid digestion, lipid transport and lipid synthesis. Lipids from food can be broken down into fatty acids, diacylglycerols and monoacylglycerols by hydrolysis of pancreatic lipase (PL, EC 3.1.1.3). These are thereafter absorbed into the hemolymph. Then, these lipids are transported through the lymphatic system to other organs in the form of lipoproteins [31]. Among the circulating lipoproteins, lipoprotein lipase (LPL, EC 3.1.1.34) is the key enzyme involved in the decomposition of TG [32]. With the hydrolysis of LPL, TG molecules are hydrolyzed to glycerol and free fatty acids [33,34]. These low-molecular substances were used for energy metabolism or stored in tissues [35]. We also analyzed the activity of two important rate-limiting enzymes in the anabolism of lipids: acetyl-CoA carboxylase (ACC, EC 6.4.1.2) and fatty acid synthase (FAS, EC 2.3.1.85) [36]. In addition, we determined the levels of ATP and NADPH, and the activity of ATP and NADPH dependent enzymes in hepatopancreas and ovary since both substances provide energy and reductive materials (H^+^) for fatty acid synthesis, respectively. In the respiratory metabolism, three main pathways (glycolysis, the tricarboxylic acid cycle, mitochondrial electron transport) contribute to ATP production [37]. Their activities are involved in three pathways, glucokinase (GK, EC 2.7.1.1), NAD^+^-linked isocitrate dehydrogenase (IDH, EC 1.1.1.41) and cytochrome C oxidase (COX, EC 1.9.3.1). These were studied along with NADPH-dependent enzyme activities, including glucose-6-phosphate dehydrogenase (G6PDH, EC 1.1.1.49) and 6-phosphogluconate dehydrogenase (6PGDH, EC 1.1.1.44) belonging to the pentose phosphate pathway. Malic enzyme (ME, EC 1.1.1.40) being a key enzyme in the citrate-malate-pyruvate shuttle, and NADP^+^-linked isocitrate dehydrogenase (IDH, EC 1.1.1.42) transforming isocitrate into α-ketoglutarate [12]. To indicate the effect of Cd on ovarian and hepatopancreas development, we further analyzed the ovarian index (OI) and hepatopancreatic index (HI) which are common indicators to evaluate tissue development in crustaceans and are associated with lipid content in tissues [24,38,39].

## Material and Methods

### Ethics Statement

The place where crabs were caught is privately owned. With the permission from the owner of the land, our study were carried out. We also confirm that the current studies did not involve endangered or protected species.

### Animals and Treatments

Adult female freshwater crabs were caught from Qinyang, Henan province in China on 29 June 2011, and were subsequently selected for examination. Before the experiment, crabs were acclimated for 3 weeks in acid cleaned glass aquaria filled with city water (dissolved oxygen 8.0-8.3 mg/l, pH 7.50 ± 0.13). During acclimatization, the temperature was kept at 20 ± 2 °C, and the aquaria were shielded using a black plastic to reduce optical disturbance. After acclimatization of 3 weeks, healthy similar-sized crabs, averaging 21.0 ± 0.7 g in weight (mean ± S. D., n = 160) were randomly divided into four experimental groups of 40 individuals each and allocated to a control group and three treatment groups exposed to 1.45, 2.9, and 5.8 mg/l CdCl_2_ corresponding to 1/160, 1/80, one-fortieth of the 96h LC_50_ according to Wang et al. [40] for 10, 15 and 20 days. Crabs in each treatment were also placed in five different tanks. To keep a constant Cd concentration, the water was renewed every two days with batches of the same concentration. During the exposure period, crabs were fed a commercial feed (Hanye, Beijing, China), containing 30% protein and 15% fat every evening (5 % animal wet weight/day). 

### Sample preparation

Haemolymph and tissues were sampled after 10, 15 and 20 days. Before sampling, crabs were anesthetized by placing them on ice for 15 min. After the whole body wet weight of crabs was weighed, heparin (400 units per kg body weight) was injected into the abdomen. A haemolymph sample of 0.2 ml was taken 5 min later, centrifuged to collect serum, and frozen temporarily at -20 °C for the subsequent measure of LPL activity and TG levels. 

After dissection of the cephalothorax, hepatopancreas and ovary were immediately sampled and their weights were recorded. The tissues were divided into several parts and stored at -80 °C for the measure of lipid, ATP, and Cd concentrations in tissues. 

Tissue pieces (0.1 g) were homogenized with ice-cold buffer (1:9, w/v) consisting of 20 mM Na_2_HPO_4_, 10 mM MgC1_2_, 1 mM phenylmethylsulfonyl fluoride (PMSF), and adjusted to pH 7.8. The homogenate was centrifuged by a refrigerated centrifuge (Eppendorf 5415R, Hamburg, Germany) for 15 min at 1,468 g at 4 °C. The clear supernatant was removed and used for the measurement of NADPH and protein levels and enzyme activities. 

### Cd determination

The analysis of Cd concentration in hepatopancreas and ovary was carried out according to the method of Ma et al. [25]. Samples (0.2 g) of every experimental condition were dried in an oven at 80 °C for 48 hours, and were then digested by 10 ml HNO_3_ (analytical grade) and 5 ml perchloric acid (analytical grade) over a hot plate at 120 °C under a reflux cap. Cd concentration in tissues was measured by electrothermal atomic absorption spectrophotometry (SHIMADZU AA-6300, Kyoto, Japan). The standard Cd solution (Shanxi Environment Protection Department, China) was used as a control of metal concentration. Cd concentration was expressed as µg/g tissue wet weight.

### OI, HI and Lipid analysis

OI and HI were defined as the percentage of tissue wet weight relative to the whole body wet weight (tissue wet weight/ body wet weight × 100). The lipid in tissues was extracted using the method of Folch et al. [41] which is very suitable for the extraction of lipids. The hepatopancreas and ovary (0.5 g) were extracted in 10 ml of methanol-chloroform (1:2 v/v) for 12 hours. The lipids dissolved in organic solvents were evaporated, and then the total lipid fraction was weighted and results were expressed as the lipid portion as the percentage of tissue wet weight.

### TG quantification and enzyme activity assay

TG levels in the haemolymph, LPL and PL activities were detected by colorimetry, using the kit (Nanjing Jiancheng Bioengineering Institute, China) according to the manufacturer’s instructions. The activities of FAS and ACC in the samples were measured by the double antibody sandwich. Elisa kits were purchased from the Immuno-Biological Laboratory (Gunma, Japan) and R&D Systems (Minneapolis, Minnesota, U.S.A.). 

### Measurements of ATP, NADPH and dependent enzyme activities

Tissue pieces were homogenized with boiling distilled deionized water (1:9, w/v). The homogenate was centrifuged for 10 min at 1,468 g at 4 °C. The supernatants were collected for the measurement of ATP concentrations using the phosphomolybdate colorimetric assay (Nanjing Jiancheng Bioengineering Institute, Nanjing, China). According to the manufacturer’s instructions, the activity of GK and COX were detected by colorimetry using kits (ATP, GK: Nanjing Jiancheng Bioengineering Institute, Nanjing, China; COX: Gemmed Scientifics Inc, Washington D.C., U.S.A.). 

The content of NADPH was measured using a spectrophotometer (SpectraMax M5, Molecular Devices Corp., San Francisco, CA, U.S.A.) by the modified method of Zhang et al. [42]. The activity of NADPH-dependent enzymes (G6PDH, 6PGDH, NADP^+^-linked IDH and ME) and NAD^+^-linked IDH was detected according to the method of Alp et al. [43] and Pierron et al. [12].

Based on the following generation of NADPH, NADH and their increased absorbance at 340 nm, the above enzyme activity was determined and expressed in nmol/min mg protein. All assays were performed under saturated substrate conditions in phosphate buffer (20 mM Na_2_HPO_4_, 10 mM MgCl_2,_ final volume, 0.8ml; pH 7.8). The final reaction mixtures were selected to provide optimal activities with homogenates and were as follows:

G6PDH: 1mM glucose-6-phosphate, 0.3 mM NADP^+^. 

6PGDH: 0.5mM 6-phosphogluconate, 0.3 mM NADP^+^. 

ME: 5 mM malate, 0.3 mM NADP^+^. 

NAD^+^-linked IDH: 0.5mM isocitrate, 0.3 mM NAD^+^.

NADP^+^-linked IDH: 0.5mM isocitrate, 0.3 mM NADP^+^.

The protein concentration in the clear supernatant fraction was determined using the method of Bradford [44], and bovine serum albumin was used as a standard.

### Data analysis

By the least-significant difference (LSD) of the one-way analysis of variance, all the data representing mean values of five independent sets of experiments and are represented by means ± standard deviation (S.D.), were used to evaluate differences between control and exposed crabs. Probability values less than 0.05 were considered significant. Statistical computations were performed with SPSS 15.0.

## Results

### Cd bioaccumulation

**Figure 1:**
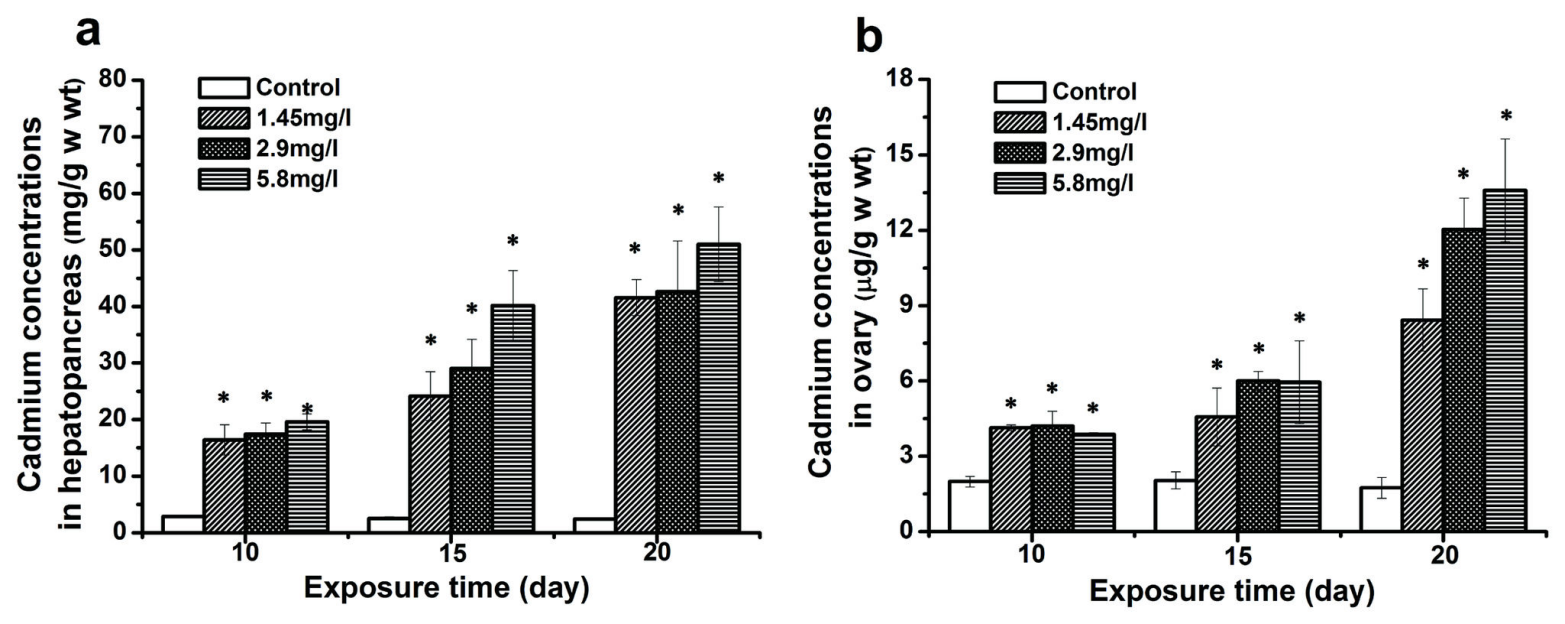
Cadmium concentrations in the tissues of control and exposed crabs. Data of hepatopancreas (a) and ovary (b) are expressed as mean ± standard deviation (n = 5). Statistical significance is denoted by * *p* < 0.05 compared to the respective control.

The results of Cd bioaccumulation in the hepatopancreas and ovary after sub-chronic Cd exposure are presented in Figure 1. Cd concentrations in exposed crabs increased significantly, and showed a distinct time- and dose-dependent pattern in both tissues. The highest Cd concentration of 50.99 ± 6.63 µg/g (w wt) was observed in hepatopancreas at 20-day of 5.8 mg/l exposure. Moreover, the Cd levels of hepatopancreas were much higher than those of the ovary at the same Cd exposure. 

### Effect of Cd on OI, HI, Lipid Content and TG Level

**Figure 2:**
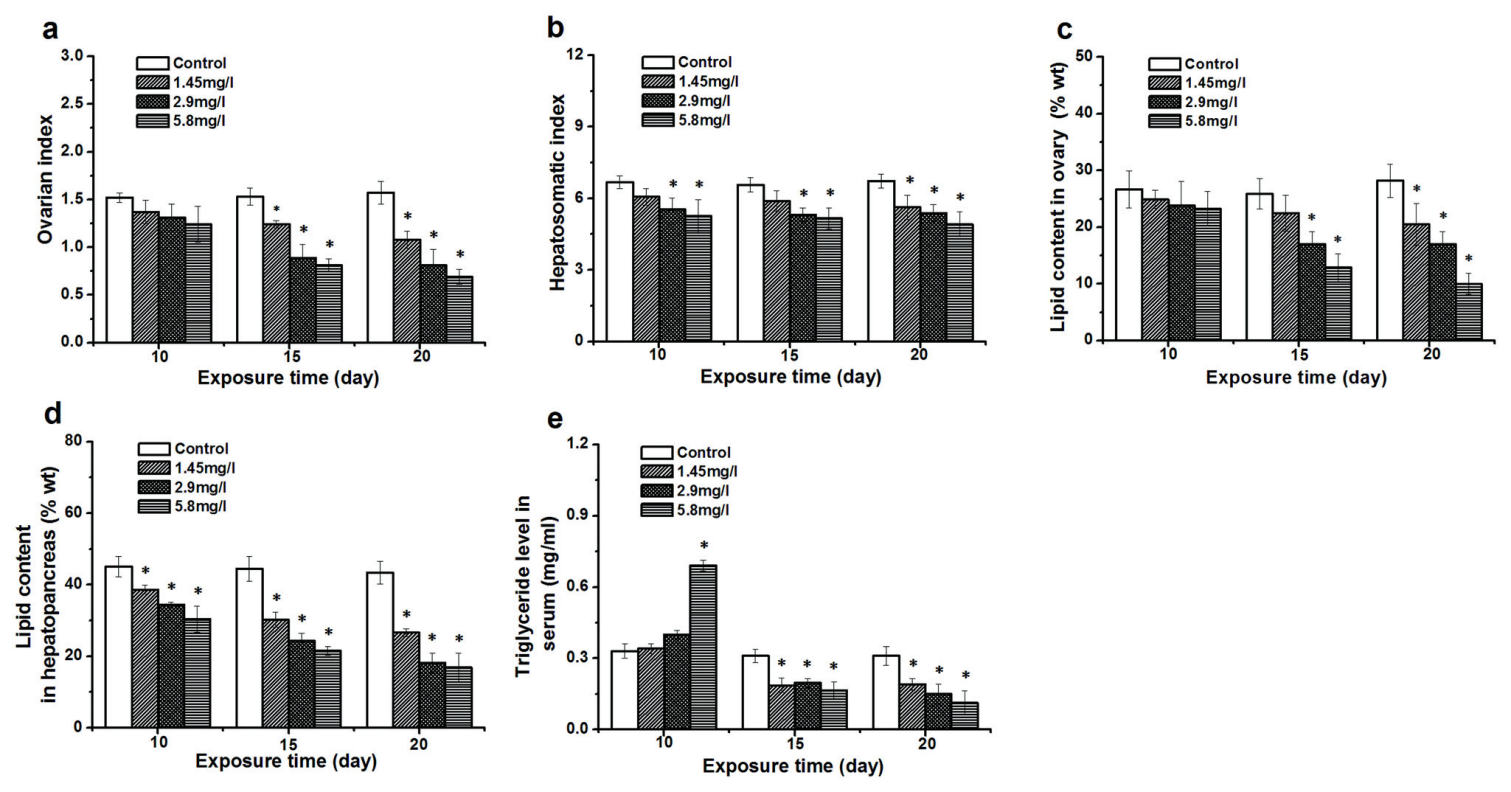
Effects of Cd on ovarian index, hepatopancreatic index, triglyceride and lipid content. Ovarian index (a), hepatopancreatic index (b), triglyceride levels in hemolymph (e) and lipid content expressed as percentage (weight of lipid/ tissue samples, FW: fresh weight) in the ovary (c) and hepatopancreas (d) for control and exposed crabs after 10, 15, and 20 days of experimentation. Data are expressed as mean ± standard deviation (n = 5). Comparison between the control and treatment groups is notified as * *p* < 0.05.

After Cd exposure, the overall lipid content of hepatopancreas in exposed crabs decreased compared to the control (Figure 2d), and a significant difference to the control (*P* < 0.05) was observed in all Cd treatment concentrations for 10, 15 and 20 days. Moreover, the HI in all exposure groups except the 1.45 mg/l Cd at 10-day exposure showed a lower level than the control (Figure 2b). However, there was no significant difference in ovarian lipid content and OI from the 10-day exposure group (Figure 2c; Figure 2a). With time, significantly lower lipid and OI levels were observed at 15 and 20 day exposures. In the experiment of the TG level in the haemolymph, significant increases were found at 5.8 mg/l Cd exposure at 10 days (Figure 2e). However, the TG level at 15- and 20-day exposure showed a significant reduction compared to the control.

### Activities of enzymes involved in lipid metabolism after Cd exposure

**Figure 3:**
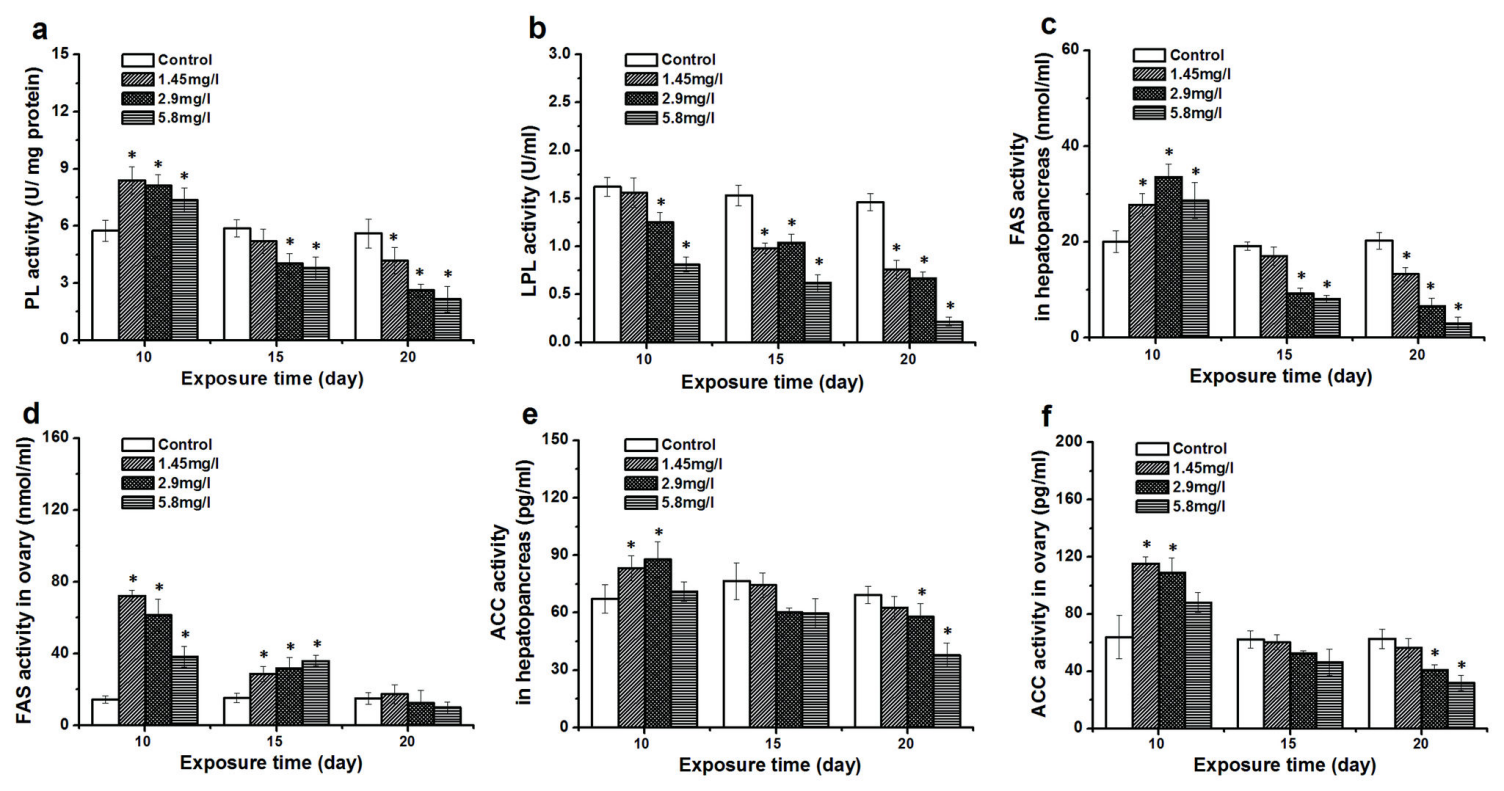
Effect of Cd on lipid digestion, lipid transport and lipid synthesis of *Sinopotamon henanense*. Activity of pancreatic lipase (PL, a), lipoprotein lipase (LPL, b) fatty acid synthase (FAS) in the hepatopancreas (c) and ovary (d) and acyl-CoA carboxylase (ACC) activity in the hepatopancreas (e) and ovary (f) of control and exposed crabs after 10, 15 and 20 days of experimentation. Comparison between the control and treatment groups is notified as * *p* < 0.05.

The activity of LPL which plays a key role in lipid transport was lower than the control, and showed a time- and dose-dependent pattern (Figure 3b). Significant differences were also observed following the exposure to 2.9, 5.8 mg/l Cd at 10-days and all Cd concentrations at 15 and 20-days. As seen in Figure 3a, the activity of PL at 10-day exposure significantly increased (*p* < 0.05). However, the PL showed a significant reduction at 15- and 20-days exposed crabs compared to the control. The activity of FAS and ACC involved in lipid synthesis at 10-day exposed crabs was higher than the controls (Figure 3b - 3f). With time both enzymes showed a lower activity, and a significant reduction of ACC activity was observed at higher concentrations at 20-days.

### ATP level and dependent enzyme activities in response to Cd exposure

**Figure 4:**
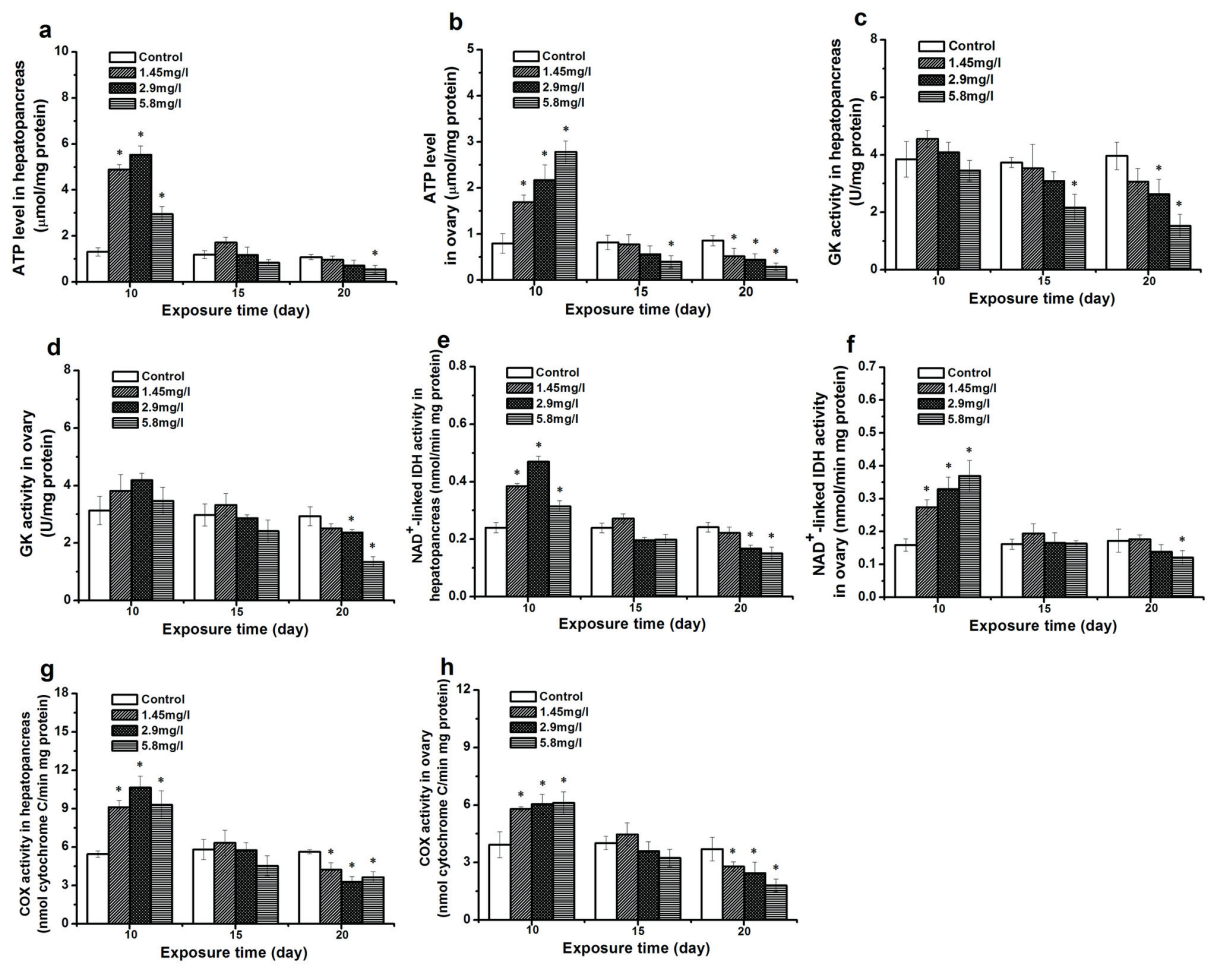
Effect of different Cd concentrations on ATP levels and dependent enzyme activities. Data are expressed as mean ± standard deviation (n = 5). Comparison between the control and treatment groups is notified as * *p* < 0.05. ATP levels: (hepatopancreas, a; ovary, b), Glucokinase (GK: hepatopancreas, c; ovary, d), NAD^+^-linked isocitrate dehydrogenase (IDH: hepatopancreas, e; ovary, f), and cytochrome C oxidase (COX: hepatopancreas, g; ovary, h).

ATP is also necessary for the synthesis of fatty acids. Figure 4 shows the results of ATP levels and the activity of enzymes involved in the mitochondrial metabolism after Cd exposure. After 10-day exposure, the ATP level in hepatopancreas and ovary of exposed crabs was significantly higher than that of the control group, and the activity of NAD^+^-linked IDH (Figure 4e; Figure 4f) and COX (Figure 4g; Figure 4h) was significantly up-regulated. However, there was no significant change observed in the GK activity in exposed crabs. Compared to the control and the 10-day exposure, the ATP level decreased and a significant change (*p* < 0.05) was observed at higher concentrations at the 15- and 20-day exposure. A similar result was found in the activity of NAD^+^-linked IDH, COX and GK.

### NADPH level and dependent enzyme activities in response to Cd exposure

**Figure 5:**
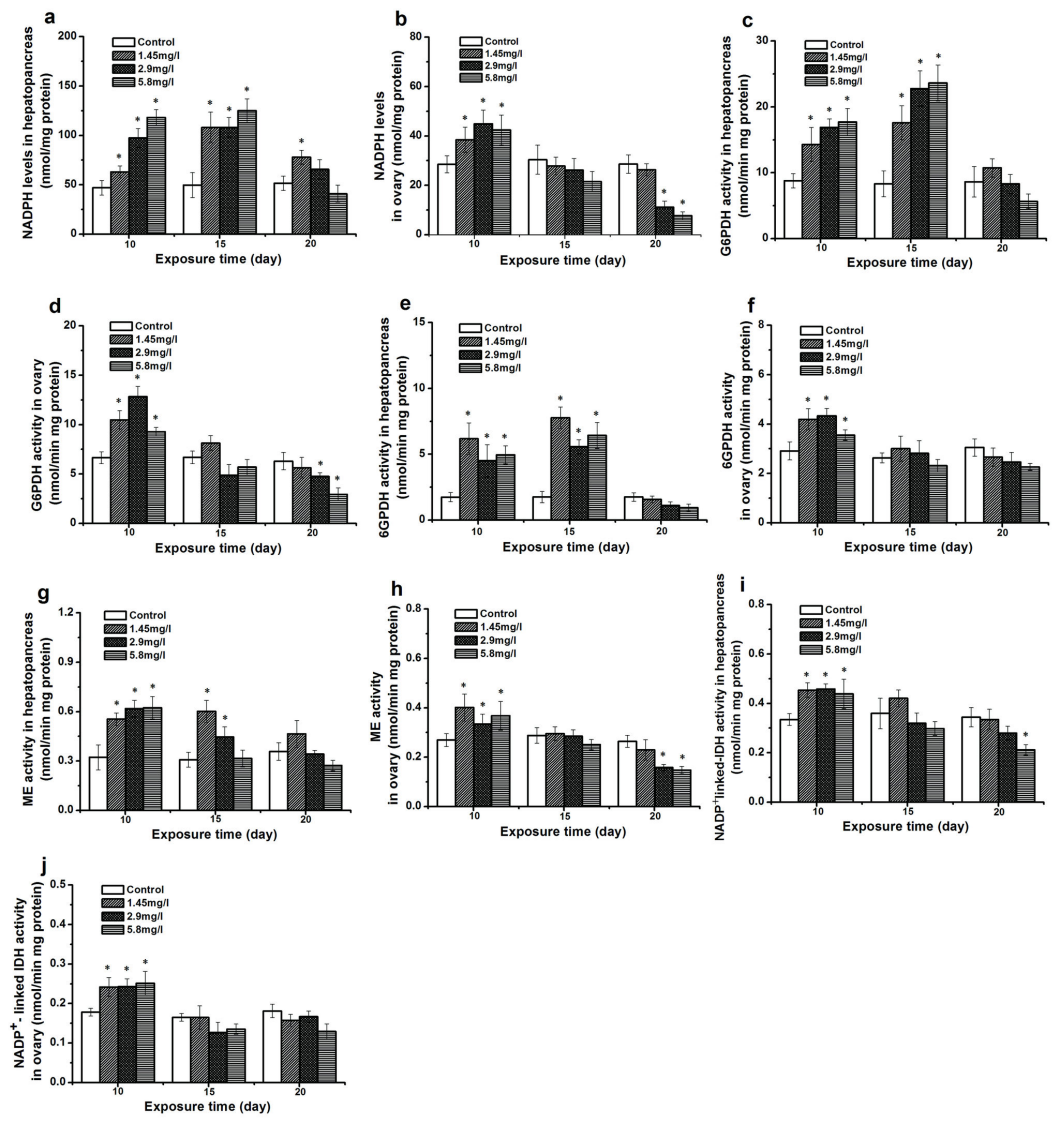
Effect of different Cd concentrations on NADPH levels dependent enzymes activities. Data are expressed as mean ± standard deviation (n = 5). Statistical significance was denoted by *p < 0.05. NADPH levels: (hepatopancreas, a; ovary, b), Glucose-6-phosphate dehydrogenase (G6PDH: hepatopancreas, c; ovary, d), 6-phosphogluconate dehydrogenase (6PGDH: hepatopancreas, e; ovary, f), malic enzyme (ME: hepatopancreas, g; ovary, h), NADP^+^-linked isocitrate dehydrogenase (IDH: hepatopancreas, i; ovary, j).

The level of NADPH, an important co-factor required in the synthesis of fatty acids, was significantly higher in hepatopancreas and ovary of crabs exposed to Cd for 10 days than in the control (Figure 5a; Figure 5b). Moreover, the activities of NADPH synthesizing enzymes were all significantly enhanced, such as G6PDH, 6PGDH, ME and NADP^+^-linked IDH (Figure 5c - Figure 5j). After 15-days exposure, NADPH levels in hepatopancreas were still higher than in the control, but no significant change was found in the ovary. However, the NADPH level at 20-day exposure decreased compared to the 10- and 15-day exposure, and a significant difference was observed in the ovary of crabs exposed to 5.8 mg/l Cd concentration. The enzyme activity associated with NADPH was down-regulated as well. 

## Discussion

### Cd bioaccumulation

We found Cd accumulation in both hepatopancreas and ovary of exposed crabs. The Cd concentration in the ovary of exposed crabs was much greater than in the control, but much lower than in hepatopancreas at the same exposure conditions. This was similar to previous studies with the shore crab *Carcinus maenas* [45] and white shrimp *Litopenaeus vannamei* [46]. The high accumulation of Cd in hepatopancreas was possibly caused by its role in detoxification. Oxidative stress is an important mechanism of Cd toxicity. Cd could alter antioxidant defense systems and stimulate the formation of reactive oxygen species (ROS) such as hydroxyl radicals and singlet oxygen [47]. ROS could cause oxidative stress by reacting with macromolecules and lead to damages, such as apoptosis and membrane lipid peroxidation [7,48]. Studies from our laboratory also found alterations in the levels of malondialdehyde (MDA) which is the final decomposition product of lipid peroxidation caused by ROS, and the activities of superoxide dismutase (SOD), glutathione peroxidase (GPx) and catalase (CAT) after Cd exposure [8,40,49,50]. In the defense against Cd-stress, one of the metal-binding proteins is metallothionein (MT), a small protein with a significant concentration of cysteine. Cysteine contains a sulphydryl group that has a high affinity to Cd. In a previous study, Ma et al. [29] found that the MT level in hepatopancreas of Cd-exposed crabs was much higher than in the control and in other tissues. Therefore, the hepatopancreas could tolerate high Cd concentrations.

### Impairment of Cd on Lipid Storage and Metabolism

Crabs that were Cd-exposed contained less lipid compared to the control, which was consistent with some previous studies in other animals such as the duck *Cairina moschata* [11], european eel *Anguilla anguilla* [12], rat [51], freshwater crayfish *Procambarus clarkii* [52], and flower shrimp *Penaeus semisulcatus* [53]. A significant reduction of lipid content in hepatopancreas of Cd-exposed crabs was observed after three experimental durations. We interpret these results with the function of lipids in the hepatopancreas. Firstly, the main function of the hepatopancreas is detoxification, which causes a higher MT level and up-regulation of antioxidant enzyme activity after Cd exposure [8,29]. This could have led to the increased energy demands in exposed crabs, which was confirmed by increased TG levels in the haemolymph, ATP levels, NAD^+^-linked IDH and COX activity after 10-day exposure. Since laboratory experiments of Xuan et al. [54] did not observe a significant reduction in carbohydrate and protein after 7 and 14 days Cd exposure, the lipid stored in hepatopancreas could be one of the main carbon sources used for ATP production. Another important function of the hepatopancreas is to provide lipids for the ovary [39]. The lipid transport mechanism existing in crustaceans such as in the crab *Pachygrapsus marmoratus* [20] and kuruma prawn [19] may be one of the reasons for a non-significant reduction of lipids in the ovary after 10-day exposure. Moreover, the lipid concentration of the hepatopancreas and ovary was closely associated with the ovarian index (OI) and hepatopancreatic index (HI). These are two common indicators to evaluate tissue development. The relationship between lipid content and HI, OI was confirmed by the change of HI, OI the change of lipids in the tissues of exposed crabs. A significant reduction of lipid levels in the tissues after Cd exposure could lead to a lower HI and OI, suggesting that hepatopancreatic and ovarian growth were seriously affected by Cd. The effect of Cd on the ovary could cause embryonic deformity and reduce hatching rates of fertilized eggs [55,56,57], affect the reproductive biology of crabs in general which is the focus of our future investigations, and may lead to the reduction of population size and other disturbances in aquatic ecosystems.

To further explore the causes of lipid impairment, we studied the activity of LP involved in lipid digestion. The results of the present study after 10-day exposure showed that LP activity was increased compared to the control, which was similar to the results of Firat and Kargin [58] and explain the increased TG levels in the haemolymph of exposed crabs. The increase of PL activity and TG level likely indicates the increased need for energy, but this trend was reversed in the reduction of lipids stored in tissues. Therefore, it was necessary to take into account the effect of Cd-exposure on lipid transport. LPL, a key enzyme involved in lipid transport in the haemolymph, was analyzed [59]. A significant down-regulation of LPL activity could also impair the lipid transport from food to tissues after 10-day exposure. This could provide another explanation for a lower lipid content.

The result of Cd on lipid metabolism demonstrated that fatty acid biosynthesis in 10-day exposed crabs could be affected by Cd. There were increased activities of two key enzymes (ACC and FAS) and the indispensable energy factors (ATP and NADPH) in the fatty acid synthesis following Cd exposure. However, an increase of lipid content in the hepatopancreas and ovary was not observed. Lucia et al. [11] explained that Cd triggered the synthesis and the mobilization of long chain fatty acids, and these were subsequently transported to the mitochondria for energy production to relieve Cd toxicity and reduce oxidative stress. Indeed, one of the principal metabolic purposes of long chain fatty acids is the production of ATP, and exposed crabs also showed a significant up-regulation of ATP levels and related enzyme activities [60]. Another explanation is that Cd-induced lipid peroxidation leads to the decomposition of polyunsaturated fatty acids in membrane systems, which could cause the impairment of the hepatopancreatic cell membrane structure [7,61]. Newly generated fatty acids could, therefore, be used for the renewal of damaged lipids [62,63]. 

In the defense against oxidative stress induced by Cd, one of the major intracellular antioxidants is glutathione (GSH) which neutralizes reactive oxygen species by the synthesis of oxidized glutathione disulfide (GSSG). The cycling of GSSG to GSH depends on NADPH [64]. NADPH as a reductant provides a necessary co-factor that plays an essential role not only in lipogenesis, but also in the entire antioxidant system [40,65]. After 10-day exposure, NADPH levels both in hepatopancreas and ovary were up-regulated. Cd seems to strongly stimulate the potential of NADPH production by increasing the activity of G6PDH, 6GPDH, ME and NADP^+^-linked IDH. According to our results on Cd affecting the lipid synthesis, we suppose here that after Cd exposure the NADPH in tissues is mainly used for GSH re-cycling.

After longer exposure (especially 20-day exposure), the impairment of Cd on lipid storage and metabolism became more serious. A higher Cd accumulation in tissues compared to the control and the 10-day exposure indicates higher energy expenditure. However, massive mobilization of lipids from tissues during Cd exposure might have caused a lower lipid level and a shortage in energy supply that was also indicated by lower TG and ATP levels. Our results showed, moreover, a reduction of the NADPH levels after 20-day exposure. These effects of Cd resulted in a reduction of Cd resistance, which could trigger the down-regulation of PL, LPL, ACC, and FAS activity. A negative impact of Cd on lipid digestion, lipid transport and fatty acid synthesis leads to an even more serious impairment of the lipid metabolism.

## Conclusions

The experimental results of the present study indicated an impairment of lipid concentrations in hepatopancreas and ovary in the Cd exposed freshwater crab, *Sinopotamon henanense*. We explain this impairment with the effect of Cd on lipid digestion, lipid transport, and energy metabolism. The lower activity of PL after a long period of Cd exposure suggests that less lipids were assimilated by crabs. The inhibition of Cd on LPL activity can cause a more serious impairment of lipid transport. Under Cd exposure, crabs showed high-energy needs, which may originate primarily from lipid consumption. However, a significant decrease of lipid levels in the tissues after a longer exposure for 15 and 20 days caused a substantial shortage in energy supplies. Moreover, the lower lipid concentrations in the ovary after Cd exposure could lead to lower HI and OI, suggesting that Cd also affected the hepatopancreatic and ovarian development of crabs. This may have ecological repercussions through its effects on reproductive fitness and subsequent population growth.
